# O3.3. REWARD PROCESSING AS A VULNERABILITY INDICATOR FOR PSYCHOSIS: RESULTS FROM A TWIN STUDY

**DOI:** 10.1093/schbul/sby015.201

**Published:** 2018-04-01

**Authors:** Mette Nielsen, Egill Rostrup, Rikke Hilker, Christian Legind, Maria H Jensen, Simon Anhøj, Brian V Broberg, Birgitte Fagerlund, Birte Glenthøj

**Affiliations:** 1 Center for Neuropsychiatric Schizophrenia Research; 2 Center for Neuropsychiatric Schizophrenia Research, & Center for Clinical Intervention and Neuropsychiatric Schizophrenia Research, Psychiatric Center Glostrup, University of Copenhagen, Glostrup University Hospital

## Abstract

**Background:**

Disturbances of the brain reward system is a common finding among patients with schizophrenia. Recent studies have found similar alterations in healthy relatives to patients with schizophrenia and reward disturbances have been suggested to be an endophenotype for schizophrenia. Here we compare brain reward activity between patients with schizophrenia, their healthy co-twin and a sample of matched healthy controls (HC). We hypothesize that patients as well as the healthy co-twins will show reward alterations compared to HC.

**Methods:**

By coupling information from The Danish Twin register to the Danish Psychiatric Central Research Register, twins with a diagnosis of schizophrenia spectrum disorder were identified and invited to participate in the study. A sample of age and gender matched HC twins were invited as well. All participants went through a diagnostic interview, and psychopathology was characterized with the Positive and Negative Syndrome Scale (PANSS) and Clinical Global Impression (CGI). Current level of function was estimated with Global Assessment of Function (GAF). The reward system was examined with a modified version of the monetary incentive delay task during functional magnetic resonance imaging (fMRI).

**Results:**

A total of 219 participants were included in the study. Of these, 162 participants completed the fMRI scanning. For statistical reasons (independent observations), only one healthy twin from each HC couple was included in the analyses, which resulted in 116 participants: 42 patients with schizophrenia spectrum disorder, 34 healthy co-twins and 40 HC. Mean age for the whole group was 40.7(10.6) and 64 (55%) were males. There were no group differences in age and sex (p>0.6).

**Psychopathology:**

Patients had a PANSS total score of 60(18.9), a CGI score of 4.1(1.5) and a GAF score of 55 (14.9). For the co-twins, PANSS-total was 36(7.1), CGI was 1.5(0.9) and a GAF was 78(11.1), whereas the HC had a PANSS total score of 31(1.6), CGI was 1(0.2) and a GAF was 86(5.6). One-way ANOVA showed a significant effect of group for PANSS total, all PANSS sub-scores, CGI and GAF. For all measures, post hoc t-tests showed significant group-differences between co-twins and HC (all p<0.03), co-twins and patients (all p<0.001) and HC and patients (all p<0.001).

Reward related fMRI activity: Whole brain group differences were analyzed in FSL by performing three pairwise comparisons: co-twins versus HC, co-twins versus patients, and HC versus patients. A corrected cluster significant threshold of P=0.05 was used.

During reward anticipation there were no group differences between co-twins and HC or co-twins and patients. Compared to HC, patients had a decreased contrast activity in the bilateral dorsolateral prefrontal cortex during anticipation of uncertain events. Likewise, patients showed decreased activity in left caudate during anticipation of loss.

During reward outcome evaluation, co-twins showed increased contrast activity compared to HC in the miss contrast and in the monetary loss contrast. Additionally, both co-twins and HC had increased contrast activity compared to patients in the miss contrast.

**Discussion:**

Although the healthy co-twins were all without clinical diagnoses, they had a subtle but significantly higher level of psychopathology and a lower level of function compared to HC. Interestingly, the healthy co-twin had an aberrant increased activity during evaluation of negative outcome compared to the HC but also compared to their diagnosed twin. This may indicate that reward abnormalities can be observed along with even subtle psychopathology and thus may serve as vulnerability indicator of psychiatric conditions, whereas these abnormalities are partly normalized by medication in the diagnosed twin.

